# A Role of Variance in Interferon Genes to Disease Severity in COVID-19 Patients

**DOI:** 10.3389/fgene.2021.709388

**Published:** 2021-09-17

**Authors:** Leonid Gozman, Kellie Perry, Dimitri Nikogosov, Ilya Klabukov, Artem Shevlyakov, Ancha Baranova

**Affiliations:** ^1^ Sackler School of Medicine, Tel Aviv University, Ramat Aviv, Israel; ^2^ School of System Biology, George Mason University, Fairfax, VA, United States; ^3^ Atlas Biomed Group Limited, London, United Kingdom; ^4^ Department of Regenerative Technologies and biofabrication, National Medical Research Radiological Center of the Ministry of Health of the Russian Federation, Obninsk, Russia; ^5^ Research Center for Medical Genetics, Moscow, Russia

**Keywords:** COVID-19, interferons, SARS-CoV-2, signaling, type I interferon, differential activity

## Abstract

The rapid rise and global consequences of the novel coronavirus disease 19 (COVID-19) have again brought the focus of the scientific community on the possible host factors involved in patient response and outcome to exposure to the virus. The disease severity remains highly unpredictable, and individuals with none of the aforementioned risk factors may still develop severe COVID-19. It was shown that genotype-related factors like an ABO Blood Group affect COVID-19 severity, and the risk of infection with SARS-CoV-2 was higher for patients with blood type A and lower for patients with blood type O. Currently it is not clear which specific genes are associated with COVID-19 severity. The comparative analysis of COVID-19 and other viral infections allows us to predict that the variants within the interferon pathway genes may serve as markers of the magnitude of immune response to specific pathogens. In particular, various members of Class III interferons (lambda) are reviewed in detail.

## Introduction

Coronavirus disease 2019 (COVID-19) is a rapidly emerging infectious disease caused by SARS-CoV-2 virus, a member of the Coronaviridae family. Since the discovery of first cases in December 2019 in Wuhan, China (World Health Organization, 2020a), the number of infected patients worldwide has been increasing logarithmically, and by December 2020 had surpassed 70 million reported cases and over 1.5 million deaths globally since the start of the pandemic (World Health Organization, 2020b).

According to WHO, mild to moderate respiratory symptoms such as fever, dry cough, upper airway congestion and sore throat are among the most common symptoms of COVID-19 (World Health Organization, 2020c; World Health Organization, 2021) which may develop over the course of 2 weeks after the exposure. However, approximately 20% of the patients develop severe or critical COVID-19 (Wu and McGoogan, 2020), characterized by pneumonia and acute respiratory distress syndrome which require hospitalization. While the overall mortality of severe COVID-19 is estimated between 1 and 4% (Ruan, 2020), in-hospital mortality in severe cases is substantially higher, reaching 28–62%, and even surpassing that in patients requiring mechanical ventilation (Weiss and Murdoch, 2020).

Multiple studies have been performed to establish the factors influencing the susceptibility to, severity and mortality of COVID-19 (Du et al., 2020; Gong et al., 2020; Vardavas and Nikitara, 2020; Verity et al., 2020). It has been shown that the severe or critical course of the disease is more likely in older adults, especially those with underlying health conditions (Centers for Disease Control and Prevention, 2020a; Garg et al., 2020), as 80% of deaths associated with COVID-19 were among adults aged 65 years or older, or those with severe comorbidities (Centers for Disease Control and Prevention, 2020b). Other proposed risk factors include smoking (Vardavas and Nikitara, 2020) and blood type (Zhao et al., 2020).

However, disease severity remains highly unpredictable, and individuals with none of the aforementioned risk factors may still develop severe COVID-19. This is particularly evident in the demographics of the disease in the United States, where even in the early stages of the outbreak 20% of the hospital admissions and 12% of the ICU admissions were attributed to people aged 20–44 years (Centers for Disease Control and Prevention, 2020b). Coupled with the first reported case of identical twins both dying of severe COVID-19 (BBC, 2020) within several days of each other, as well as data obtained in pilot studies on the heritability of COVID-19 symptoms (Williams et al., 2020), it strongly suggests that inherited DNA variants play a significant role in the severity of the disease.

### *ACE2* and Viral Entry

The rapid rise and global consequences of the novel coronavirus disease 19 (COVID-19) has brought the focus of the research on the possible contribution of the host factors to patient response and outcome of exposure to the virus. SARS-coronavirus 2 (SARS-CoV-2), the pathogenic cause of the disease, relies on similar mechanisms of cellular entry as SARS-CoV; namely, the SARS-CoV-2 receptor angiotensin-converting enzyme II (ACE2) and the serine protease TMPRSS2, which facilitates the priming of spike (S) protein for viral entry (Hoffmann et al., 2020; Zhou et al., 2020).

Early on during the rise of the pandemic, there was a hope that variations in the *ACE2* gene may account for resistance or susceptibility to COVID-19 in different populations. It was shown that populations in East Asia had higher allele frequencies in the expression quantitative trait loci (eQTL) in *ACE2,* which might have led to increased expression of the enzyme (Cao et al., 2020). Likewise, given that *ACE2* is located on chromosome X, a hope was expressed for explaining the gender differences in response to the disease, namely the fact that men were disproportionately more susceptible to the SARS-CoV-2 virus (Majdic, 2020).

Early studies attempting to connect variation in *ACE2* and *TMPRSS2* loci on the risks of contracting COVID-19 in any form, so far, have produced inconclusive results, ranging from single SNP associations uncovered in small cohorts (Latini et al., 2020) to the lack thereof. The latter, possibly, is due to the dual role of ACE2, which serves both as an entry into the wells and a lung-protective molecule (Dalan et al., 2020; Nagy et al., 2021).

A large study by Lopera Maya et al. (2020) used the Lifelines cohort data to analyze the association between the variants within *ACE2* or *TMPRSS2* loci and cardiac, pulmonary, renal and other quantitative phenotypes, which are also pertinent to COVID-19. Despite the large sample size and wide variety of variants and quantitative phenotypes examined, no statistically significant association was detected. The study found, however, an intriguing association between the use of angiotensin receptor blockers (ARBs) and non-steroidal anti-inflammatory drugs (NSAIDs) and variants at the *ACE2* and loci. As the diseases associated with the use of these medications are commonly comorbid with COVID-19 (Lopera Maya et al., 2020), these findings may eventually prove their relevance to COVID-19 severity. Drugs based on the inhibition or blockage of TMPRSS2 protease are undergoing clinical trials as a therapeutic option for COVID-19 treatment (Abbasi et al., 2021). Thus, there remains continued interest in studying the *ACE2* and *TMPRSS2* genes as determinants of susceptibility to SARS-CoV-2.

### ABO Blood Group and Disease Severity

After the SARS-CoV outbreak in Hong Kong in 2003, researchers showed a relationship between the blood type of the participants that had been exposed to the virus and the chance of contracting infection (Cheng et al., 2005). It appeared that exposed individuals with blood group O phenotype were less susceptible to SARS-CoV infection, even while previous studies had shown that they had increased susceptibility to infection with either Norwalk virus or *H. pylori* (Cheng et al., 2005). The association of ABO blood groups with the risk to contract coronavirus disease has also been noted during the current pandemic. Retrospective study conducted in China showed that patients with blood group O had a significantly lower risk of infection and hospitalization with SARS-CoV-2, while patients with blood group A had a higher risk of infection and hospitalization (Li et al., 2020).

Further research has both confirmed this association and shed more light on it. Retrospective studies conducted in various regions of China, New York, Italy, Spain, and Turkey have shown a higher odds ratio for being infected with SARS-CoV-2 for patients with blood type A phenotype as well as a lower one for blood type O patients when (Focosi, 2020). A genome wide association study conducted in Italy and Spain regarding the genetic associations between individuals infected with COVID-19 and respiratory failure, confirmed that patients with blood group A had a higher risk of COVID-19-induced respiratory failure while blood group O granted patients a protective effect (The Severe Covid-19 GWAS Group, 2020). Two loci with a genome-wide significance were found, namely, the rs11385942 insertion-deletion GA at locus 3p21.31 and the rs657152 A at locus 9q34.2. The association signal at 9q34.2 coincided with the *ABO* locus, further implicating the connection between patient’s ABO blood group and the course and danger of the disease (The Severe Covid-19 GWAS Group, 2020). Later, a multicenter study performed in Canada showed that COVID-19 patients with blood group A or AB are at increased risk for requiring mechanical ventilation and prolonged ICU admission compared with patients with blood group O or B (Hoiland et al., 2020), thus, supporting *in silico* GWAS results by patient’s ward observations.

Interestingly, the viral infectivity features due to the ACE2 receptor binding, and due to contribution of the blood antigens may be related to each other. In case of SARS-CoV, the presence of anti-A antibodies, which is a characteristic of groups O and B, inhibits the adhesion of the virus to the ACE2 receptor (Guillon et al., 2008). It is tempting to speculate that this finding may be directly relevant to SARS-CoV-2 as well, given that these findings are consistent with the host response to other viruses such as measles and HIV (Arendrup et al., 1991; Preece et al., 2002) and a trend in increased efficiency of the transfusion of the convalescent plasma from O or B group donors (Hacibekiroğlu et al., 2021).

### 3p21.31 Locus

In addition to findings reported from Italy/Spain (The Severe Covid-19 GWAS Group, 2020), a separate study comprising 3,199 hospitalized patients with COVID-19 and control individuals was released by the COVID-19 Host Genetics Initiative in which the region on chromosome 3 was the only major genetic risk factor for severe symptoms after SARS-CoV-2 infection and hospitalization at the genome-wide level (The COVID-19 Host Genetics Initiative, 2020). It is not clear which specific gene within the region identified on chromosome 3 is associated with COVID-19 severity. In particular, this region harbors *CXCR6* and *CCR1* genes, encoding important chemokines, which control the movement of immune cells and are critical for the innate immune system to function properly (Sokol and Luster, 2015). Another gene of this region, *SLC6A20*, encodes a protein that functions as a proline transporter expressed in alveolar cells, kidney and small intestine (SIT1), which is known to bind to ACE2 (Camargo et al., 2009; Vuille-dit-Bille et al., 2015; Wang et al., 2020). Notably, the entire fragment may have been inherited from Neanderthals, entering the human genome during the period of interbreeding between the two groups (Zeberg and Pääbo, 2020), and is differentially represented in human population samples.

### Other Loci

Interferons (IFNs) are central to antiviral immunity. Previously it was shown that type I IFN deficiency in the blood could be a hallmark of severe COVID-19 and provide a rationale for combined therapeutic approaches (Hadjadj et al., 2020).

Additional studies have shown the importance of other loci in determining the genetic susceptibility of hosts to COVID-19, particularly in determining which patients are susceptible to severe manifestations of the illness. A recent study of patients with life-threatening COVID-19 pneumonia looked at thirteen loci involved in either the TLR3 or IRF7 dependent pathways for the amplification of type I IFN, and found that 3.5% of patients had deleterious variants (pLOF) in eight of the tested loci, underlining how impairment of the production of type I IFNs can lead to critical SARS-CoV-2 infection (Zhang et al., 2020). Similarly, a recent study of critically-ill COVID-19 patients in the United Kingdom used Mendelian randomization to show the potential for a causal relationship between the *IFNAR2* gene which codes for a receptor subunit in interferon signalling and disease severity, and concluded likewise that the administration of interferons may aid in patient recovery, while acknowledging that it is as yet unclear when during the course of the illness they may provide therapeutic benefit (Pairo-Castineira et al., 2021). Moreover, the study was able to replicate the results of a previous study on the 3p21.31 locus, and a transcriptome-wide association study that they performed on the patient pool showed that the variant in oligoadenylate synthetase (*OAS*), rs10735079 affected expression of *OAS3*, which codes mediator involved in the degradation pathway of double-stranded RNA, which is itself involved in the replication pathway of coronaviruses (Pairo-Castineira et al., 2021).

### *ACE2* as an Interferon-Responsive Gene

Notably, in humans, the *ACE2* belongs to a family of interferon-stimulated genes (ISGs), which typically serve to promote a complex and uniform response to an infection-related spike in interferon levels (Schneider et al., 2014). Moreover, in human lung epithelial cells, the levels of *ACE2* mRNA are co-correlated with that of TMPRSS4, and many immune response pathways, including proinflammatory interleukins and IFI16 (Wruck and Adjaye, 2020). Specifically in human nasal epithelial cells, *ACE2* expression is upregulated by type I (IFN-α and IFN-β) and type II (IFN-γ) interferons (Ziegler et al., 2020). The efficacy of this process may be affected by genetic variations in any part of this cascade. However, in this review, we would like to bring attention to a particular component of the interferon response, which has been massively implicated in the natural and therapeutic outcomes for other viral diseases, namely, the *IFNL4* gene. This gene encodes a type III interferon IFN-λ4, capable of blocking some of the interferon signaling, resulting in poor response to HCV treatment with IFN (Sung et al., 2017). Notably, type III IFNs have been proposed as more viable therapeutic option for prevention and treatment of COVID-19 than type I IFNs, particularly because they cause fewer and milder systemic side effects (Muir et al., 2014; Prokunina-Olsson et al., 2020). It has also been shown that Type III IFNs are highly effective at preventing the viral spread from the nasal epithelium to the upper respiratory tract (Klinkhammer et al., 2018). Additional studies may be warranted to explore the mechanisms of interaction between SARS-CoV-2 and type III interferons, and to estimate how they are affected by the status of the IFNL4 gene.

It is anticipated that influence of ACE2 in COVID-19 сan potentially be exploited for the rational design of effective SARS-CoV-2 therapeutics (Ni et al., 2020; Barros et al., 2021).

### Role of Human Interferons in Viral and Non-Viral Liver Disease

Interferons are a class of cytokines that mediate the host immune response to infection by viral and non-viral pathogens (Crosse et al., 2017; Bogdan et al., 2004; Seliger et al., 2008). They are categorized into three types based on their protein sequence (O’Brien et al., 2014) (Table 1). Type I interferons are rapidly produced when viral envelope glycoproteins, CpG DNA, or dsRNA interact with host cell receptors such as mannose receptors, toll-like receptors, and cytosolic receptors (Malmgaard, 2004). Type 1 interferons can directly activate natural killers (NKs), antigen-presenting dendritic cells as well as CD4 and CD8 T cells (Hervas-Stubbs et al., 2011). All type I interferons signal through a common receptor interferon alpha receptor (IFNAR). The IFNAR induces the Janus activated kinase-signal transducer and activation of transcription (JAK-STAT) pathway that control a large collection of genes through regulated expression of various signaling intermediaries (Guan et al., 2014; Messina et al., 2016; Olex et al., 2016).

**TABLE 1:** Classification of interferons.

Interferon (IFN)Type	Receptor type	Protein structure	Genes	Gene location	Tissue expression pattern
Type I	IFN α receptor that consists of IFNAR1 and IFNAR2 chains	α−helix	IFN-α 2a and 2b	Chr. 9	Leukocytes, macrophages, endothelial cells, tumor cells, keratinocytes, and mesenchymal cells
			IFN-b		Fibroblasts, endothelial cells, macrophages, and epithelial cells
			IFN-ω		T lymphocytes
			IFN-ε		Cerebral tissues
			IFN-κ		Not known
Type II	IFNGR consisting of IFNGR1 and IFNGR2 chains	Core of six α− helices and an extended unfolded sequence in the C-terminal region	IFN-γ	Chr. 12	T and Natural Killer cells
Type III	Receptor complex consisting of IL10R2 and IFNLR1 chains	Structurally similar to the IL-10 family, despite functionally being an IFN	IFN-λ		Dendritic cells and macrophages

Type I interferons are rapidly produced when viral factors, such as envelope glycoproteins, CpG DNA, or dsRNA, interact with cellular pattern-recognition receptors (PRRs), such as mannose receptors, toll-like receptors (TLRs), and cytosolic receptors (Malmgaard, 2004). These interferons directly activate natural killers (NKs), antigen-presenting dendritic cells (DC) as well as CD4 and CD8 T cells (Hervas-Stubbs et al., 2011). In T cells, the signaling through the IFNAR is critical for the acquisition of effector functions (Kole et al., 2013).

Type II interferons are represented by pleiotropic Th1-type cytokine interferon-γ. The IFN-γ is induced in response to a variety of cytokines, including interleukin-2 (IL-2), IL-18, Type I IFNs alpha/beta, or by stimulation through T cell receptors (TCRs) or NK cell receptors (Malmgaard, 2004). Similar to Type I interferons, IFN-γ stimulates the JAK/STAT pathway. In addition, a number of other pathways, including MAP kinase, PI3-K, CaMKII, and NF-kappaB cross-talk with JAK-STAT signaling to fine-tune the multifaceted effects of IFNγ, which are exerted in a gene- and cell type-specific manner (Gough et al., 2008).

The type III family of interferons are comprised of IFN-λ1, IFN-λ2, and IFN-λ3 or IL-29, IL-28A, and IL-28B, respectively (Kotenko et al., 2003; Gad et al., 2009; Lin and Young., 2014; O’Brien et al., 2014). These interferons signal through a receptor complex composed of the IFN-λR1 chain (also known as IL-28RA) and the IL-10R2 chain, which is also a part of the receptor complexes for IL-10, IL-22, and IL-26. (Sheppard et al., 2003; Gad et al., 2009; Donnelly and Kotenko, 2010; Lopušná et al., 2013).

In 2013, a new member of the interferon λ (lambda) family, IFN-λ4, was described which signals through the IFNλR1 and IL-10R2 receptor chains (Hamming et al., 2013). The IFN-λ4 is encoded by the gene IFNL4, whose expression has been shown to be upregulated in response to HCV infection, but not to HBV infection (Estep et al., 2014).

Recent studies point that IFN dysregulation may be the key to determining COVID-19 pathogenesis (Andreakos and Tsiodras, 2020; Lopez et al., 2020; Meffre and Iwasaki, 2020). There is evidence that the response to class I interferons in COVID-19 is impaired. In the blood of patients with severe COVID-19, amounts of class I IFNs are much lower when compared to that of patients infected with highly pathogenic influenza viruses. Nevertheless, in the lungs, in bronchoalveolar lavage in some seriously ill COVID-19 patients, local induction of IFN genes becomes noticeable. A dysregulated interferon response is considered part of the immunomodulatory strategies used by some coronaviruses, including SARS-CoV-2 (Acharya et al., 2020). Nevertheless, a recent pan-ancestry exome-wide association study of rare genetic protein-coding variants and various t COVID-19 outcomes didn’t find any significant associations in any of the 13 interferon pathway genes (Kosmicki et al., 2021).

Since the beginning of the pandemics, interferons were repeatedly seen as a viable option for boosting the host’s defences against SARS-CoV-2. Indeed, early evidence suggests that SARS-CoV-2 may be more susceptible to pretreatment with type I IFNs, even more so than SARS-CoV (Lokugamage et al., 2020; Sallard et al., 2020). Later, in human intestinal cells, the treatment with interferon-lambda and respective responses showed efficiency at controlling SARS-CoV-2 replication (Stanifer et al., 2020). In this light, a renewed attention was paid to type III interferons, which have being tested as therapeutics in COVID-19 outpatients, either with no success (Jagannathan et al., 2021) or with limited virological response detected (Feld et al., 2021). The difference in outcomes of the interferon-lambda based therapeutics may be explained by the varied presence of neutralizing IFNL3 autoantibodies pre-existing in patients that later develop severe COVID-19 (Credle et al., 2021).

### The *IFNL4* Locus

**FIGURE 1:**
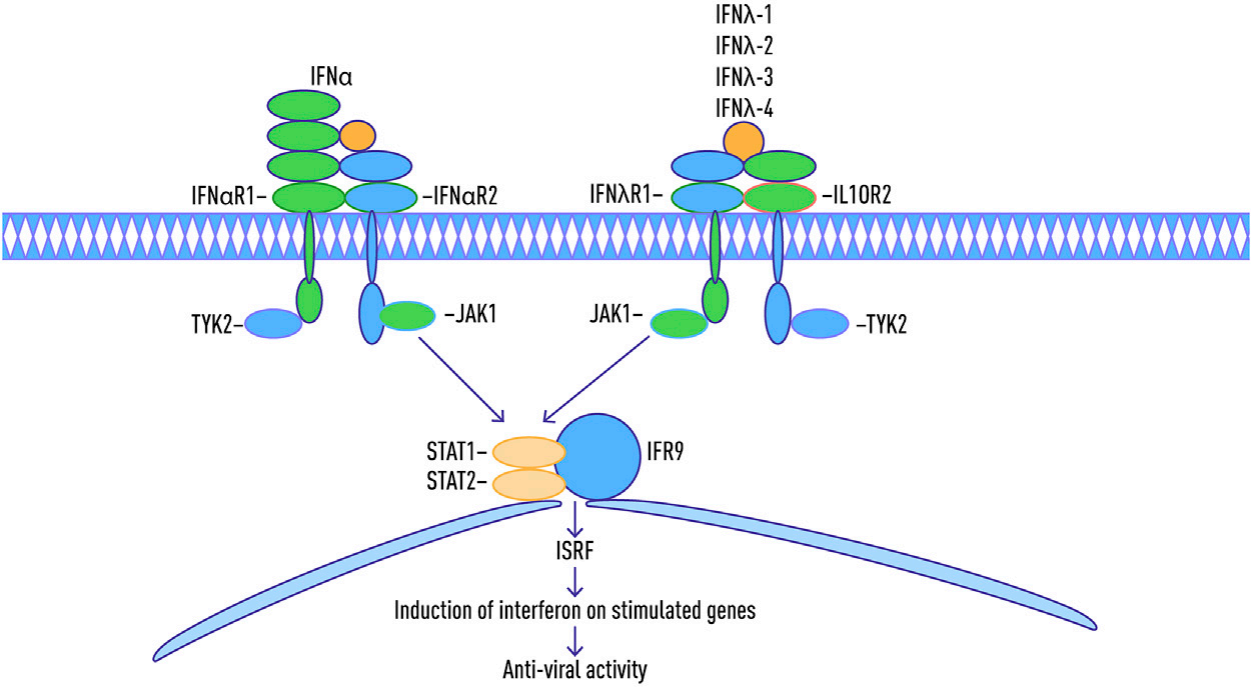
Location of common SNPs in IFNL4 Locus on Chromosome 19, and the map of IFNL4 exons. Adapted from: Stephen M. Laidlaw and Lynn B. Dustin, 2014, with changes.

The *IFNL4* gene is located on chromosome 19q13, just over 1 kb upstream of, and in the same orientation as, the gene encoding IFN-λ3 (Figure 1). It is extremely conserved in all mammals, indicating its functional importance (Key et al., 2014). The ancestral allele of *IFNL4,* contains a guanine residue at position 342 of the coding sequence (referred to as “ΔG”). It encodes a functional IFN-λ4 peptide.

The *IFNL4* locus is known to contain a number of medically relevant single nucleotide polymorphisms (SNPs). One of these, rs368234815 or ss469415590 (TT) is characterized by the substitution of the G nucleotide with two thymine residues (TT) resulting in a nonsense mutation. As a result, IFN-λ4 can be generated only by individuals, who carry the ΔG allele, To date, the majority of the studies of *IFNL4* locus have been performed in the context of hepatitis C virus (HCV). In contrast to other IFNs, expression of IFNλ4 is associated with decreased clearance of HCV in the human population; by contrast, a natural frameshift mutation that abrogates IFNλ4 production improves HCV clearance. The ΔG allele is associated with adverse outcomes of infection and interferon-based treatments (Amanzada et al., 2013; Franco et al., 2014 AIDS; Aka et al., 2014; Nozawa et al., 2014; Jouvin-Marche et al., 2014; Stättermayer et al., 2014) while the TT allele is associated with the spontaneous clearance of HCV and interferon responsiveness. It is presently the strongest known host factor for predicting clearance of HCV (O’Brien et al., 2014). Another SNP, known as rs12979860, located within the intron of IFNL4 gene, is closely linked to the rs368234815 allele and is significantly associated with sustained viral response (SVR) in HCV patients (Younossi et al., 2012).

In a genome-wide association study published in 2009, the presence of a rs12979860 with a “C” allele was strongly associated with spontaneous viral clearance and treatment response (Ge et al., 2009). Patients who were homozygous for the presence of “C” allele had a greater than 2-fold increase in rates of SVR as compared to patients with heterozygosity of this locus (C/T allele combination) and homozygous state T/T (Younossi et al., 2012; Meissner et al., 2014; Stättermayer et al., 2014). In addition to increased SVR rates, patients homozygous for C allele (C/C) were more likely to demonstrate spontaneous clearance of HCV (Thomas et al., 2009). Additionally, the presence of SVR-promoting rs12979860 allele of *IL28B* locus was associated with lower baseline inflammation and possible suppression of apoptosis in peripheral blood mononuclear cell (PBMCs) evaluated during early phase of the treatment as compared to the presence of deleterious allele (Younossi et al., 2012). These findings were confirmed in the 2013 GWAS performed in 13 international multicenter study sites (Duggal et al., 2013).

Interpretation of these findings relies on the proximity linkage of rs12979860 (*IL28B*) to rs368234815 (*IFNL4*) that is functionally responsible for effects of both variants. Due to shorter average size of haplotype blocks in individuals of African ancestry, rs368234815 is more strongly associated with HCV clearance in these ethnicities, whereas in Europeans and Asians it performs similarly to rs12979860 (Prokunina-Olsson et al., 2013).

Non-functional rs368234815-TT allele is specific for humans and is common in all human populations. In HapMap collection, it is detected in 93% of Asians genomes, 68% of European genomes, and 23% of Africans genomes (Prokunina-Olsson et al., 2013).

Similar frequencies of distribution were observed in the 1000 Genomes Project samples: the TT allele is present in 89.8–95.2% of Chinese genomes, in 68.9% of European genomes and in 29.3% of African genomes (The 1000 Genomes Project Consortium, 2015).

Linkage disequilibrium between rs368234815-TT allele and rs12979860-C allele results in the same frequencies distribution pattern for the latter genetic variant: C allele is present in 89.8–95.2% Chinese genomes, in 69.1% of European genomes and in 33.1% of African genomes (The 1000 Genomes Project Consortium, 2015). Frequency of the rs12979860 C/C genotype in IFNL4 gene was significantly lower in COVID-19 patients (*p* < 0.001) (Saponi-Cortes et al., 2021).

Linkage between these two genetic variations rises from Africans (R^2^ = 0.8318) through Europeans (R^2^ = 0.9815) and is absolute in Chinese populations (R^2^ = 1.0) (Machiela and Chanock, 2015).

It is theorized that it emerged right before the onset of the “out-of-Africa” migration and was immediately supported in its spread by positive selection in European and Asian populations resulting in the high frequency observed today (Key et al., 2014).

It is most likely that the selective force that was driving elimination of IFN-λ4 in a majority of human populations was an exposure to a certain pathogen (or pathogens), most likely a virus. However, this pathogen is unlikely to be HCV, which is known for its relatively slow progress toward symptomatic phase (Fan et al., 2016). There is also no association between *IFNL4* polymorphisms and HBV susceptibility or natural clearance (Fan et al., 2016) and the advantages or disadvantages of IFN-λ4 expression in case of infection with a majority of non-HCV non-HBV viruses remain unknown.

**FIGURE 2:**
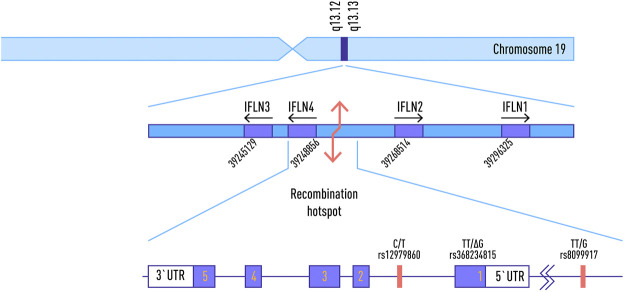
Schematic map of Jak-STAT pathway during an immune response with type 1-3 interferon antiviral activity.

A number of recent studies showed that IFN-λ4 possesses strong antiviral activity toward HCV and coronaviruses (Hamming et al., 2013; Prokunina-Olsson et al., 2013). When over-expressed in a hepatoma cells, *IFNL4* induces STAT1/STAT2 phosphorylation and expression of interferon-stimulated genes (Prokunina-Olsson et al., 2013; O’Brien et al., 2014; Randall and Goodburn, 2008; Ank et al., 2006) (Figure 2). Interestingly, when studied against either HCV or coronavirus (HCoV-229E and MERS-CoV) challenges tested in either human ciliated airway epithelial cell (HAE) or hepatocyte cultures, the antiviral activities of recombinant IFNλ3 and IFNλ4 were similar (Hamming et al., 2013). Another recent comparative study of Type III interferons, this time performed using transcriptome sequencing, also failed to reveal any crucial differences between particular members of this family, with the majority of the identified genes being similarly regulated in hepatocytes as well as airway epithelial cells (Lauber et al., 2015). Hence, it looks like the differences in mode of action for various IFN-λ may be due to their direct binding to some cellular or viral targets rather than to the transcription programs they stimulate.

IFNLR is expressed at relatively high levels in respiratory epithelial cells, and mice treated with IFN-λ prior to infection with human metapneumovirus (HMPV) develop lower viral titers and reduced inflammatory responses. On the other hand, Ifnlr1 −/− mice exhibit increased susceptibility to respiratory viral infections, including influenza virus, HMPV, respiratory syncytial virus, and SARS coronavirus (Lazear et al., 2015).

In contrast, Prokunina-Olsson proffered the hypothesis that functional IFN-λ4 protein may compete with the IL28B/IFN-λ3 receptors and apparently cause a pre-activation of the interferon-dependent genes, thus, reducing overall responsiveness to Type I and III interferon (Prokunina-Olsson et al., 2013). This hypothesis is a good agreement with previous findings that SVR-promoting alleles are associated with lower baseline inflammation (Younossi et al., 2012). Notably, in a small study of rs12979860 allele distribution in COVID-19 patients and controls, the “C” allele, previously associated with favourable HCV outcomes and lower baseline inflammation, showed association both with higher susceptibility to coronavirus and with poorer outcomes of SARS-CoV-2 disease (Agwa et al., 2021).

There is some evidence that the polymorphisms in IFNλ4 may influence outcomes of non-HCV non-coronavirus types of acute and chronic infections. In particular, solid-organ transplant recipients homozygous for the active, ancestral rs368234815 allele (ΔG) are more susceptible to CMV replication, especially in absence of antiviral prophylaxis (Egli et al., 2014; Manuel et al., 2015). Another study showed that the same allele is associated with increased susceptibility to AIDS-related CMV retinitis (Bibert et al., 2014).

Findings related to IFN-lambda gene variants in patients with HIV infection remain controversial. One study showed that, in Caucasian populations, the CC genotype of rs12979860, which is associated with favourable HCV outcomes, is also associated with spontaneous control of human immunodeficiency virus (HIV) viremia (Machmach et al., 2013). In cohorts of African Americans these findings, however, were not replicated (Sajadi et al., 2011; Salgado et al., 2011). In a study of Real and co-authors, pseudogenized allele rs368234815-TT that protects against infection with HCV was also associated with decreased likelihood of HIV-1 infection in male intravenous drug users [odds ratio (OR): 0.3; *p* = 0.006], and this association was not modified by the genotype of CCR5 (Real et al., 2015). Another recent study of rs368234815 variant showed that carriers of its active, ancestral variant ΔG have a higher occurrence of AIDS-defining illnesses and lower CD4 T-cell counts (Machmach et al., 2015). These results suggest that genetic susceptibility to HCV and HIV-1 infection may share a common molecular pathway (Real et al., 2015).

It should be noted that the relationships between pre-existing HIV infection and COVID-19 are still unclear (Centers for Disease Control and Prevention, 2020b), most likely due to limited cross-testing (Jones et al., 2020). While the use of protease inhibitors such as lopinavir and ritonavir had a positive effect on patients with MERS-CoV, recent research suggests that in patients with SARS-CoV-2 these compounds do not work (Jones et al., 2020; Jothimani et al., 2020). Attempts to utilize known anti-HCV treatments in COVID-19 wards had failed in a similar way (Huang et al., 2020).

Nevertheless, recent events have unequivocally shown that the coronaviruses, in general, and the SARS-CoV-2, in particular, should be regarded as yet another evolutionary driver for the fine-tuning of human interferon response to existing and emerging pathogens. Population frequencies of *IFNL4* and other interferon-encoding gene variants may reflect a sum of past exposures to the various pathogens, and the subsequent bottlenecks which may or may not be related to epidemic events.

## Conclusion

Genetic resistance to severe viral diseases shares common molecular pathways for various viral infections. Interferon expression pathways in host cells make a crucial contribution to the proinflammatory response to infectious agent appearance. The presence of some variants in the loci of interferon gene sequences reduces the natural immunity, and stimulates a susceptibility to severe viral disease.
